# MiR-126-5p Promotes Tumor Cell Proliferation, Metastasis and Invasion by Targeting TDO2 in Hepatocellular Carcinoma

**DOI:** 10.3390/molecules27020443

**Published:** 2022-01-10

**Authors:** Yang Ai, Sang Luo, Ben Wang, Shuai Xiao, Yefu Wang

**Affiliations:** The State Key Laboratory of Virology, College of Life Sciences, Wuhan University, 299 BaYi Road, Wuhan 430065, China; aiyang9311@163.com (Y.A.); 2018102040017@whu.edu.cn (S.L.); 2019202040019@whu.edu.cn (B.W.); xiaoshuai825@hotmail.com (S.X.)

**Keywords:** hepatocellular carcinoma, MiR-126-5p, TDO2, tryptophan metabolism, RIP

## Abstract

TDO2 is a key enzyme in the kynurenine metabolic pathway, which is the most important pathway of tryptophan metabolism. It has been shown that miRNAs are involved in cell metastasis through interaction with target mRNAs. In this study, we found 645 miRNAs that could be immunoprecipitated with TDO2 through the RNA-immunoprecipitation experiment. miR-126-5p was selected as the research target, which was also confirmed by dual-luciferase reporter assay. Through qRT-PCR analysis, it was verified that the overexpression of miR-126-5p promoted the expression of TDO2, PI3K/AKT and WNT1. Meanwhile, it was verified that overexpression of miR-126-5p can promote intracellular tryptophan metabolism by HPLC. We also verified the effects of miR-126-5p on cell proliferation, migration, and invasion by cck-8, cell colony formation and trans-well assay in both HCCLM3 cells and HepG2 cells. In vivo experiments were also conducted to verify that miR-126-5p promoted tumor formation and growth via immunohistochemical detection of cell infiltration and proliferation to generate markers Ki-67, BAX, and VEGF. In conclusion, our results suggest that miR-126-5p is a biomarker and a potential new treatment target in the progression of HCC via promoting the expression of TDO2.

## 1. Introduction

HCC (hepatocellular carcinoma) is a serious threat to health and one of the most common gastrointestinal malignancies, with the third highest mortality rate [1]. About 383,000 people in China die of liver cancer every year, which accounts for 51% of all liver cancer deaths in the world [2]. Although a variety of strategies have been used to treat HCC, such as liver transplantation, liver resection, radiofrequency ablation, and transarterial chemoembolization, the prognosis of HCC is still poor because of tumor invasion, frequent intrahepatic spread, and extrahepatic metastasis [3]. Therefore, it is necessary to find candidate therapeutic targets, and to improve the quality of life of HCC patients.

TDO2 (tryptophan 2,3-dioxygenase) is a key enzyme in the tryptophan-kynurenine metabolic pathway. The human TDO2 protein contains 406 amino acids; it is encoded by TDO2 gene on chromosome 4, with a length of 65,669 bp, including 12 exons and 11 introns. [4]. In humans, TDO2 is highly expressed in liver cancer and is involved in immune tolerance of liver cancer cells. TDO2 interacts with AhR (aryl hydrocarbon receptor) and CYPlAl (cytochrome P4501Al), thereby participating in the occurrence and development of various tumors [5]. Unlike IDO (indoleamine 2,3-dioxygenase), which is another tryptophan-degrading enzyme that participates in the kynurenine pathway, TDO2 is not regulated by IFN-γ (interferon-γ), but only by glucocorticoids, and it can only metabolize L-tryptophan [6]. The role of TDO2 in cancer biology has been demonstrated, which has led to extensive research into its mechanism of action [7]. TDO2 is indeed upregulated in many solid tumors, leading to immune evasion, and it is associated with poor prognosis in patients [8]. Moreover, TDO2 inhibitors are a hotspot in anticancer drug development [9]. Previous studies have shown that inhibitors of TDO2 can prevent tumor immune resistance and promote tumor rejection [10]. Therefore, it is also worth studying to find other small molecules that can regulate TDO2 expression.

MiRNAs (microRNAs) are a class of noncoding small molecular RNAs with a length of about 18–22 nt and a high degree of conservation. MiRNAs play a role in negative regulation of the mRNA expression of target genes in the development of tumors by completely or partially complementing the target gene mRNA’s 3’-UTR region [11]. More than 20,000 kinds of miRNAs have been found so far; among them, nearly 1,000 kinds of miRNAs are related to tumors, but there are also other undiscovered signaling pathways or new miRNAs involved in tumor metastasis [12]. Moreover, there are more complex synergistic or antagonistic effects between these signaling pathways and miRNAs [13].

In recent years, many studies have focused on the abnormal expression of miRNAs in HCC. It has been shown that the dysregulation of miRNAs is related to the development and progression of HCC as miRNAs play a role as oncogenes or tumor suppressors [14,15,16,17]. In addition, miRNAs participate in the metastasis process of tumor cells by regulating various signaling pathways, such as PI3K (phosphatidylinositol-3-hydroxykinase)/AKT (protein kinase B) and TGF-β (transformin-g growth factor-β) signaling pathways [18,19,20]. Dysregulated miRNAs are considered useful biomarkers for clinical diagnosis and prognosis of cancer, as well as potential therapeutic targets for cancer therapy [21]. Targeting these miRNAs may be a novel approach in the treatment of HCC. Currently, the therapeutic applications of miRNAs mainly involve miRNA inhibition and miRNA replacement therapy [22]. In addition, several recent studies have shown that miRNAs are effective prognostic predictors of HCC [23,24].

MiRNA-126-5p is an miRNA that has attracted much attention. The low expression of miR-126-5p was associated with tumor invasion and metastasis in most cases [25]. Some studies screened and identified partial target genes of miR-126-5p. For example, miR-126-5p can inhibit the proliferation, invasion, and migration of HCC cells by targeting EGFR (epidermal growth factor receptor) [26]. Miao et al. [27] found that miR-126-5p could inhibit the migration of breast cancer cells by directly targeting CNOT7 (ccr4-not transcription complex subunit 7). Gu et al. [28] found that the negative regulation of CDK6 (cyclin-dependent kinase) expression by miR-126-5p could inhibit the proliferation and migration of esophageal cancer cells. In addition, Meng et al. [29] found that miR-126-5p could negatively regulate the expression of BCAR3 (breast cancer anti-estrogen resistance protein 3) in endometriosis, enhance the migration and invasion of endometriosis cells, and promote the occurrence of endometriosis. In colon cancer, miR-126-5p has been shown to inhibit tumor growth by regulating a subunit of the target gene PI3K [30]. However, miR-126-5p expression is significantly increased in patients with non-relapsed gastric cancer and decreased in patients with relapsed gastric cancer [31]. This suggests that a single miRNA can regulate multiple target genes, thereby forming a complex regulatory network during the occurrence and development of tumors. Furthermore, the role and expression level of the same miRNA may differ in different tumors, and it may even differ in different types or stages of the same tumor [32]. At present, there have been few reports on the relationship between miR-126-5p and HCC. Moreover, no studies have yet confirmed the relationship between miR-126-5p and TDO2.

In this study, we attempted to investigate the biological role of miRNA-126-5p in the progression of liver cancer and its interaction with TDO2 to provide reference for the diagnosis and treatment of liver cancer. HCCLM3 cells (human hepatocellular carcinoma cells) were used to verify the relationship between miRNA-126-5p and TDO2 targeting, so as to explore the effect of miRNA-126-5p-based TDO2 targeting on the invasion, migration, and apoptosis of liver cancer cells and further study the underlying mechanism.

## 2. Results

### 2.1. Search for miRNAs That Can Regulate TDO2

To find miRNAs that can regulate TDO2, RNA-immunoprecipitation (RIP) experiment was applied to obtain the “protein–RNA” interaction complex by immunoprecipitation with antibodies against TDO2. Then RNA sequencing was performed to screen out the RNA that could bind to TDO2.

**Figure 1:**
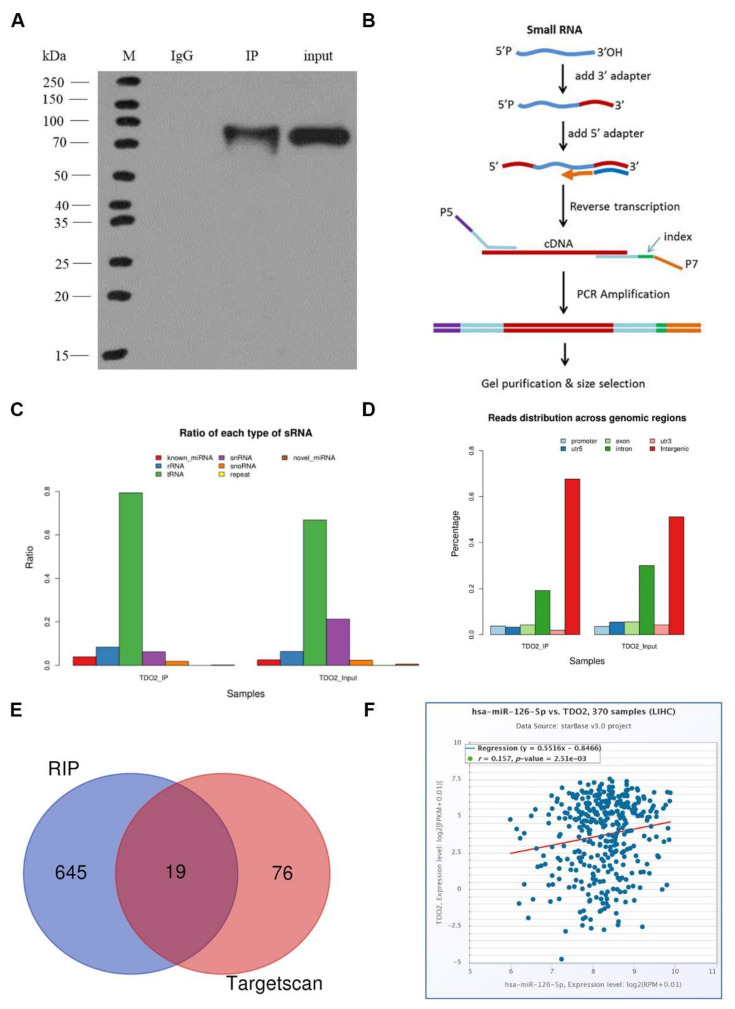
MiRNAs that can regulate TDO2 expression were screened out by RIP. (**A**) TDO2-Flag protein bands enriched in IP and input groups. (**B**) cDNA library construction process. (**C**) sRNA classification annotated statistics. (**D**) The histogram of the distribution of the sample in different regions of the reference genome. (**E**) Venn diagrams of miRNAs predicted by Target Scan and analyzed by RIP. (**F**) The regression equation of the correlation between miR-126-5p and TDO2.

As shown in Figure 1A, TDO2-Flag bands were detected in the IP group and the input group, indicating that Flag antibodies were enriched in the TDO2-Flag protein in the system. The construction schematic diagram is shown in Figure 1B. To ensure that each unique sRNA has a unique annotation, known miRNAs were compared with several different annotations at the same time. The order of detection priority (rRNA > tRNA > snRNA > snoRNA > repeat > gene > novel RNAs) was used to classify and statistically annotate the small RNAs (Table 1 and Figure 1C). Clean reads of each sample were screened for sRNA within a certain length range for subsequent analysis. A bowtie was used to locate the length-screened sRNA onto the reference sequence and analyze the distribution of small RNA on the reference sequence (Table 2 and Figure 1D). Using RIP, we found 645 known miRNAs and 138 novel miRNAs that can bind to TDO2. We then searched for miRNAs containing conserved 8 mer, 7 mer, and 6 mer sites that matched the 3’-UTR region of TDO2 through TargetScan 7.2 to find the corresponding biological targets. We found 95 corresponding miRNAs on TargetScan 7.2. After statistical analysis using Venn plot, 19 common miRNAs were found (Table 3 and Figure 1E). Finally, miR-126-5p, which had most reads in the samples and had no reported effect on TDO2 in the literature, was selected for our study. The 645 miRNAs were mentioned in the Supplementary Material. Finally, the regression equation of the correlation between miR-126-5p and TDO2 obtained in the Starbase database suggests that they are positively correlated (*r* = 0.157).

### 2.2. miR-126-5p Can Directly Bind to TDO2

**Figure 2:**
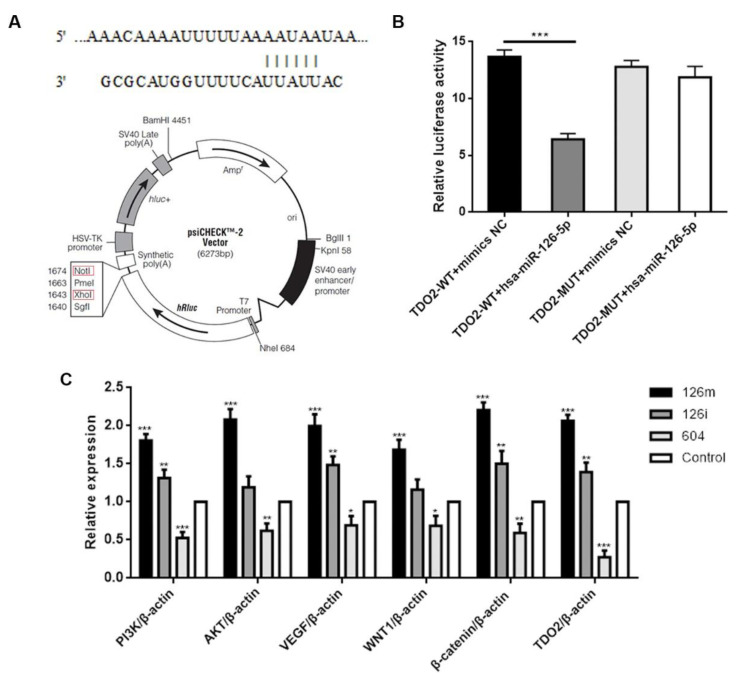
MiR-126-5p binds to TDO2 and influences the expression of the related signaling pathway proteins. (**A**) Atlas of plasmid (psiCHECK-2 Vector) was used for dual luciferase assays. (**B**) After transfection with miR-NC or miR-126-5p in HEK293T cells, the relative luciferase activity of mutant or wild-type TDO2 3′-UTR was detected. (**C**) Effect of 126m (miR-126-5p mimics), 126i (miR-126-5p inhibitors), 604 (siRNA-TDO2) on signaling pathways and TDO2 expression. All experiments were conducted at least three independent times, and all the values were presented as mean ± standard error of the mean (SEM). * *p* < 0.05, ** *p* < 0.01, *** *p* < 0.001.

Subsequently, to explore whether miR-126-5p can directly bind to the 3′-UTR region of TDO2, we cloned the wild-type and mutant binding target of miR-126-5p into psicheck2 vector (Figure 2A). XhoI and NotI were selected as insertion sites. We co-transfected 293T cells with miR-NC or miR-126-5p and psicheck2 vector. Then after sequencing verification, the base sequence in the vector was consistent with the expected sequence, which confirmed the completion of the construction of the double luciferase vector (Supplementary Materials). As shown in Figure 2B, the relative luciferase activity of wild-type TDO2 vector was significantly reduced after transfection of miR-126-5p. However, when the mutant TDO2 was co-transfected with miR-126-5p, the reduction in luciferase activity was completely eliminated. In conclusion, these results suggest that TDO2 can interact with miR-126-5p.

### 2.3. Effect of miR-126-5p and TDO2 on Regulation of Signal Path Proteins

The potential mechanism of miR-126-5p was further studied. We first found that the upregulation of miR-126-5p (group 126m with transfected miR-126-5p mimics) significantly increased the relative expression levels of PI3K, AKT, WNTI1, and β-catenin, in contrast, inhibitors of miR-126-5p (group 126i with transfected miR-126-5p inhibitors) appeared to decrease their expression levels. Moreover, their influence on VEGF trends were also as described above. We also found that miR-126-5p mimics significantly increased the expression of TDO2, while miR-126-5p inhibitors had almost no effect on the expression of TDO2 (Figure 2C). Meanwhile, the group 604 with transfected siRNA-TDO2 showed the lowest expression level. These results suggest that the PI3K/AKT pathway and the WNT pathway may be involved in miR-126-5p-related promotion of proliferation and metastasis. These deserve further analysis.

### 2.4. MiR-126-5p Promoted HCCLM3 Cell Proliferation, Migration and Invasion In Vitro

**Figure 3:**
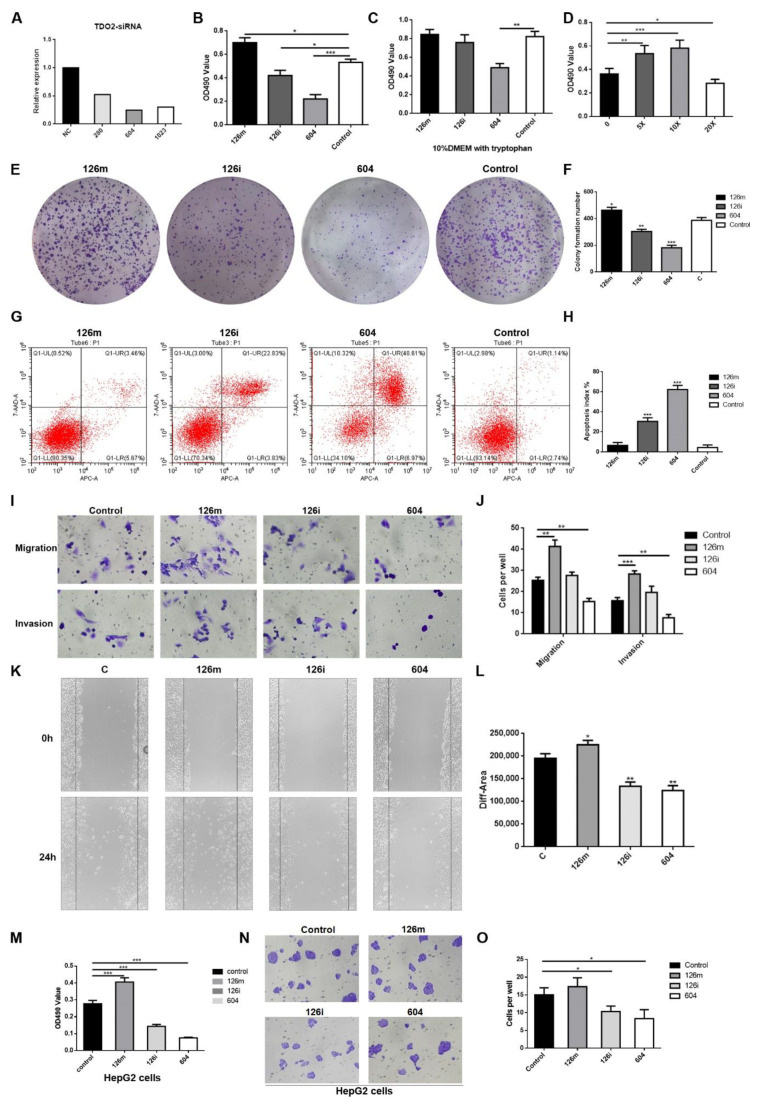
MiR-126-5p can significantly promote the proliferation, migration, and invasion of HCCLM3 cells, and inhibit apoptosis. (**A**) Selection of the most effective siRNA of TDO2. (**B**) Effect of miR-126-5p on HCCLM3 cells growth determined by CCK-8 assays. Group 126m represents HCCLM3 cells transfected with miR-126-5p mimics; group 126i represents those treated with miR-126-5p inhibitor; group 604 represents siRNA-TDO2; NC represents the negative control group. (**C**) The transfected HCCLM3 cells recovered some activity after replacing the medium with 10× tryptophan. (**D**) The effects of media containing different multiples of tryptophan on cell activity. Group 0 represents the cells that were changed with normal medium after transfection. The n× group represents the medium that contained n times as much tryptophan as the original medium after transfection. (**E**,**F**) Colony formation assays in HCCLM3 cells. (**G**,**H**) Effect of miR-126-5p on HCCLM3 cell growth determined by flow cytometry. (**I**,**J**) Transwell assays results in HCCLM3 cells. (**K**,**L**) Wound healing assays showing the miR-126-5p-related promotion of the migratory abilities of HCCLM3 cells. (**M**) Effect of miR-126-5p on HepG2 cells growth determined by CCK-8 assays. (**N**,**O**) Transwell assays results in HepG2 cells. All experiments were conducted at least three independent times, and all the values were presented as mean ± SEM. * *p* < 0.05, ** *p* < 0.01, *** *p* < 0.001.

Our results suggest that TDO2 was a direct target of miR-126-5p and the expression of TDO2 was significantly decreased after treatment with siRNA of TDO2 (Figure 3A). As verified by qPCR, TDO2-siRNA (604) had the most obvious inhibitory effect on TDO2. To determine whether miR-126-5p can affect the malignant phenotype of HCCLM3 cells, we examined the effects of miR-126-5p overexpression or downregulation on cell proliferation, invasion, apoptosis, and angiogenesis. The analysis of CCK-8 results shows that miR-126-5p mimics had the best proliferation promotion effect compared with the control group, while TDO2 inhibitor (604) group showed an inhibitory effect (Figure 3B). However, after transfection, the replacement of medium containing high concentration of tryptophan restored the inhibitory effect of miR-126-5p inhibitor and reversed the inhibitory effect of 604 to a certain extent (Figure 3C). The content of tryptophan in the replaced medium was determined (Figure 3D); given that the medium containing 10 times tryptophan (10×) had the best effect on cell proliferation, we chose it to study the reversal effect. Clone formation assays confirmed that miR-126-5p mimics enhanced the proliferative ability (Figure 3E,F). Flow cytometry results also confirmed the results shown above (Figure 3G,H). Transwell (Figure 3I,J) and scratch-wound (Figure 3K,L) experiments confirmed that miR-126-5p mimics significantly promoted the invasion and migration of HCCLM3 cells, while the results of miR-126-5p inhibitor and 604 were the opposite. Similarly, we conducted CCK-8 and trans-well experimental verification on HepG2 cells, which was another HCC cell line. The results were consistent with those obtained in HCCLM3 cells. As shown in Figure 3M–O, miR-126-5p mimics promoted proliferation and migration of HepG2 cells, while miR-126-5p inhibitor showed inhibition. In conclusion, our data support that miR-126-5p mimics promote the malignant characteristics of HCCLM3 cells by improving the expression of TDO2.

### 2.5. Effect of miR-126-5p on Tryptophan Metabolism in Cells

**Figure 4:**
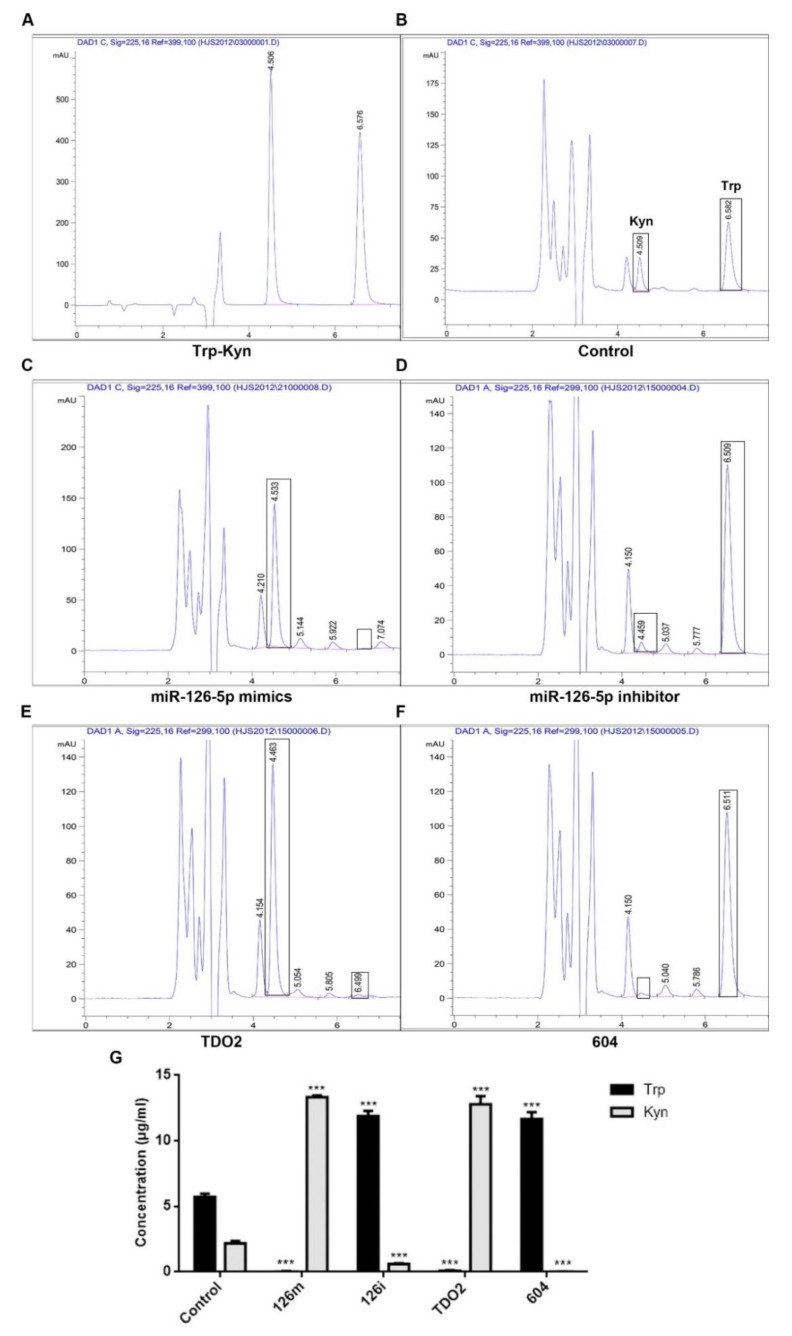
The contents of tryptophan and kynurenine in each group after transfection were determined by HPLC. The *X*-axis is time (min), and the *Y*-axis is mAU. The peak at 4.5 min is kynurenine, and the peak at 6.5 min is tryptophan. (**A**) The peak diagram of tryptophan and kynurenine standard. (**B**) The peak diagram of DMEM medium containing 10% FBS. (**C**–**F**) The peak diagram of cell transfection with miR-126-5p mimics, miR-126-5p inhibitor, TDO2 and siRNA-TDO2(604). (**G**) Statistical results of the peak area of each elution peak. Data are shown as mean ± SD, *n* = 3; *** *p* < 0.001 vs. control.

As shown in Figure 4A–G, after transfection, the contents of tryptophan and kynurenine in the medium which were detected by HPLC (high performance liquid chromatography) were widely different. All tryptophan in the cells transfected with miR-126-5p mimics and TDO2 was metabolized to kynurenine. However, the cells transfected with miR-126-5p inhibitor and siRNA-TDO2(604) group contained large amounts of tryptophan and almost no kynurenine. This further confirms that miR-126-5p promoted the expression of TDO2 and promoted the metabolism of tryptophan in cells, thus affecting cell proliferation and metastasis.

### 2.6. miR-126-5p Promotes Tumorigenesis of HCCLM3 Cells In Vivo

**Figure 5:**
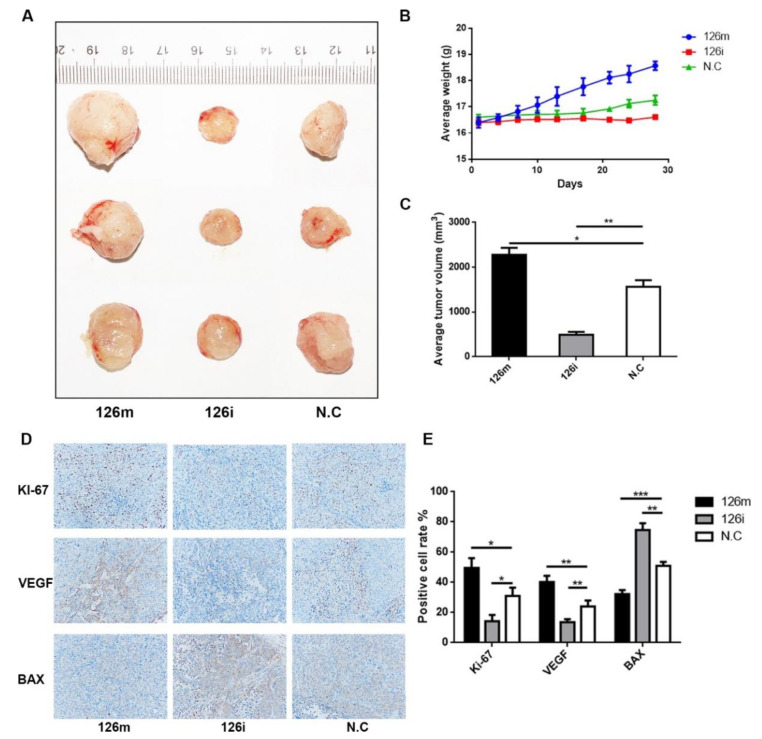
Verification of promotion effect of miR-126-5p in nude mice transplantation experiment. (**A**) BALB/c nude mice were taken for xenotransplantation, and the size was measured by the ruler. (**B**) The body weight of nude mice showed significant intergroup difference due to tumor size. (**C**) The volume of anatomized subcutaneous xenograft tumors was measured and analyzed by Student’s *t* test. (**D**,**E**) Representative Ki-67, BAX, and VEGF immunohistochemical staining micrographs of xenograft tumors. Ki-67 and VEGF were significantly increased in the group 126m, while BAX was significantly reduced. The 126i group showed the opposite trend. Data are shown as mean ± SD, *n* = 3; * *p* < 0.05, ** *p* < 0.01 and *** *p* < 0.001 vs. NC.

HCCLM3 cells were subcutaneously implanted into the ventral side of nude mice, and the effect of miR-126-5p on tumorigenicity was studied by measuring the weight and volume of the transplanted tumors in nude mice. The mice were weighed every 3 days, starting from 7 days after the implantation. As shown in Figure 5A–C, the 126m group had significantly promoted tumor growth, and the effect showed a gradient increase with the increase of dose. However, the 126i group showed a certain inhibitory effect. Immunohistochemical staining results (Figure 5D,E) showed that the percentage Ki-67-positive cells in the 126m group were the highest among the three groups, so was the VEGF-positive cells in the 126m group. The expression level of Bax was the highest in the 126i group. These results are consistent with those from our in vitro experiments and suggest that miR-126-5p has a promoting effect on tumor growth.

## 3. Discussion

MiRNAs can exist in a variety of forms, and they have independent promoters and regulatory sequences. They are widely involved in biological processes, such as cell proliferation, apoptosis, and differentiation; moreover, they are closely related to the occurrence and development of tumors [33]. Abnormally expressed miRNAs (e.g., miR-300, miR-130b) have been found to be closely related to the occurrence, progression, and metastasis of liver cancer by regulating EMT and acting as tumor suppressor or oncopromoter genes [34]. MiRNAs can directly interact with cytokines in the Wnt signaling pathway. For example, miR-504 can bind to Wnt3A mRNA and downregulate the expression of Wnt3A, thereby inhibiting the invasion and migration of HCC cells through the positive regulation loop of Wnt/PRC1 [35].

In this study, by qPCR, we confirmed that miR-126-5p upregulated the expression levels of Wnt1 and β-catenin, and that the Wnt signaling pathway was involved in the regulation of miR-126-5p. Cui et al. [36] proposed that miR-129-3p is a tumor suppressor gene in liver cancer, which can inhibit the growth and invasion of liver cancer cells through the targeted regulation of Aurora-mediated PI3K/Akt signaling pathway. Similarly, we confirmed that miR-126-5p upregulated the expression of PI3K and AKT, and indirectly verified that the PI3K/AKT signaling pathway was involved in the regulation of miR-126-5p.

To study miRNAs that can regulate the expression of TDO2, we chose RIP experiment to find miRNAs that can bind to TDO2. According to the experimental results, we found a total of 645 known miRNAs in the database. To more conveniently study the interaction between miRNA and TDO2, we made predictions on TargetScan 7.2. Finally, through Venn analysis, 19 miRNAs were identified. Of them, miRNA-126-5p had the highest expression level in the sample, although it had not been reported in the literature. Thus, we chose it for our study. Subsequently, we also verified the interaction between miR-126-5p and TDO2 by double luciferase experiment. The lucifase activity was decreased after co-transfection of wild-type TDO2 and miR-126-5p mimics, suggesting that miR-126-5p may be involved in regulating TDO2 expression. However, after co-transfection of mutant TDO2 and miR-126-5p mimics, luciferase activity remained unchanged, which further confirmed that miR-126-5p could bind to the 3’UTR region of TDO2, thus targeting its expression.

Although we confirmed the interaction, their specific influencing mechanism needs to be further analyzed. The results of qPCR indicated that the expression of TDO2 increased after transfection with miRNA-126-5p. This indicates that miRNA-126-5p plays a positive regulatory role in the expression of TDO2. In addition, we know that TDO2 is involved in human tryptophan metabolism, and that it can metabolize tryptophan to kynurenine [19,37]. Therefore, we indirectly evaluated the effect of miRNA-126-5p on TDO2 by detecting the contents of Try and Kyn in the transfected groups with mimics and inhibitors of miRNA-126-5p, TDO2 and TDO2-siRNA (604). After detection by HPLC, it was found that tryptophan was completely metabolized to kynurenine in the miRNA-126-5p- and TDO2-transfected groups. In contrast, tryptophan was barely metabolized in the miR-126-5p inhibitor group and the siRNA-TDO2 (604) group.

Through the CCK-8 test, wound healing test, and colony formation test, we confirmed that miR-126-5p promoted the proliferation of HCCLM3 cells and HepG2 cells. Flow cytometry also verified the effect of miR-126-5p on HCCLM3. The upregulation of TDO2 expression also significantly promoted the proliferation and metastasis of HCCLM3. We also verified the promoting effect of miR-126-5p on the invasion and migration of HCCLM3 cells and HepG2 cells through a transwell experiment. Subsequently, in rescue experiments, by changing the medium to that containing higher concentrations of tryptophan after administration, the promotion of proliferation and metastasis was partially eliminated. It was demonstrated that sufficient amounts of tryptophan were also able to reverse the inhibition of miR-126-5p inhibitors and siRNA-TDO2.

Next, qPCR was used to detect the expression levels of cell migration-related factor (VEGF), PI3K/AKT and key proteins of Wnt signaling pathway in each group. After transfection with miR-126-5p, the expression levels of both VEGF and signaling pathway proteins were increased, while the inhibitors showed inhibitory effect. These findings indirectly support the promoting effect of miR-126-5p on tumor cells.

In the in vivo experiment, we chose to inject miR-126-5p agomir and its inhibitor antagomir directly into the tumor, so as to see the difference of tumor formation more intuitively. We found that after agomir injection, the tumors became significantly larger than NC group. Through imaging and immunohistochemistry, we found that miR-126-5p agomir significantly promoted tumor migration and proliferation. Ki-67 (reflecting cell proliferation) and VEGF (reflecting cell migration) inhibition were observed after knockdown of miR-126-5p. These results were consistent with in vitro experiments.

In this study, we first verified the regulatory effect of miR-126-5p on TDO2. Subsequently we confirmed both in vivo and in vitro experiments that TDO2 and miR-126-5p mimics can promote the proliferation, invasion and migration of HCC cells, while knockdown TDO2 and miR-126-5p inhibitors can inhibit the tumor progression of HCC cells. Therefore, we can say that miR-126-5p, as a TDO2 regulatory factor, affects the proliferation, invasion and migration of HCC cells. Until now, the role of TDO2 in the formation and development of different tumor cells has not been determined [38]. While our anti-cancer ideas are based on the tryptophan metabolic pathway. Specifically, we found that miR-126-5p expression affected tryptophan metabolism through targeting TDO2 expression and showed a significant association between tumor growth and tryptophan metabolism. In the future, we can combine miR-126-5p inhibitors, TDO2 inhibitors, IDO inhibitors and other tryptophan metabolic enzymes (e.g., tryptophan side-chain oxidase enzyme) to develop anticancer drugs. At the same time, we will further study the mechanism of tryptophan metabolism in tumor development.

## 4. Materials and Methods

### 4.1. Cell Culture

293T and HCCLM3 cell lines were purchased from the National Typical Culture Preservation Center (preservation center of Wuhan University, Wuhan, China). All cell lines were confirmed to be free of Mycoplasma contamination, as determined by PCR and culture methods. The species origin of each cell line was confirmed via PCR. The identity of each cell line was authenticated via STR (short tandem repeat) profiling. These cell lines were cultured in dulbecco’s modified eagle medium (DMEM) (Hyclone, Logan, UT, USA) containing 10% fetal bovine serum (FBS) (AusgeneX, Queensland, Australia), 100 units/mL penicillin, and 100 μg/mL streptomycin (Hyclone, Logan, UT, USA) at 37 °C in a humidified atmosphere with 5% CO_2_. The medium was replaced every 2–3 days, and the cells were subcultured when the cell fusion rate reached 80%–90%.

### 4.2. Reagents

The following commercially available reagents were also used: anti-Flag tag (Cali-Bio, California, CA, USA, cat: CB100145M, 1:1000 dilution), anti-BAX (Servicebio, cat: GB11007-1, 1:50 dilution, Wuhan, China), anti-Ki67 (Abcam, cat: Ab16667, 1:200 dilution, Cambridge, MA, USA), anti-VEGF (Servicebio, cat: GB11007-1, 1:100 dilution, Wuhan, China).

The following commercially available reagents were also used: SDS sample buffer (Sinopharm Group Chemical Reagent Co., Ltd., Beijing, China, cat: 30166428), 30% acrylamide (Biosharp, cat: BL513b, Hefei, China), Trise-Base (Biofavor Biotech, Biofroxx, cat: 1115GR500, Wuhan, China), Glycine (Biofavor biotech, Biofroxx, cat: 1275GR500, Wuhan, China), methyl alcohol, NaCl, KCl, Na2HPO4.12H2O, KH2PO4, acetic acid, Tween-20 (Sinopharm Group Chemical Reagent Co., Ltd., Beijing, China), protein markers (Thermo Fisher Scientific, Waltham, MA, USA), protein markers (Thermo Fisher Scientific, Waltham, MA, USA), and PVDF membranes (Millipore, MA, USA), micrON has-miR-126-5p agomir (RIBOBIO, cat: miR40000444-4-5, Guangzhou, China), micrOFF has-miR-126-5p antagomir (RIBOBIO, cat: miR30000444-4-5, Guangzhou, China).

### 4.3. MiRNAs, siRNAs, and Plasmid Constructs

DNA transfection and RNA interference were performed via Lipofectamine 3000 (Thermo Fisher Scientific, Waltham, MA, USA) in accordance with the manufacturer’s instructions. Small-interfering RNA (siRNA, 50 nmol/L) was used in all experiments for 293T and HCCLM3 cell lines, and miRNA mimics were transfected at concentrations of 50 nmol/L, as indicated in the respective assays. In addition, siRNAs against TDO2, the control siRNA, miR-126-5p mimic, control mimic, miR-126-5p inhibitor, and control inhibitor were from Sangon Biotech (Shanghai, China). The miR-126-5p mimics sense sequence was: 5′-CAUUAUUACUUUUGGUACGCG-3′; the anti sense sequence for miR-126-5p mimics was 5′-CGUACCAAAAGUAAUAAUGUU-3′. The miR-126-5p inhibitor sense sequence was: 5′-CGCGUACCAAAAGUAAUAAUG-3′. The siRNA sense sequences for TDO2 were as follows: 5′-AUCAUAACUCAUCAAGCUUAUTT-3′ (280); 5′-CGUGAUAACUUCAAAGGAGAATT-3′ (604); 5′-GAUGACCAAAUGGAGAUAUA-ATT-3′ (1023). The antisense sequences for TDO2 were as follows: 5′-AUAAGCUUGAU-GAGUUAUGAUTT-3′ (280); 5′-UUCUCCUUUGAAGUUAUCACGTT-3′ (604); 5′-UUAUAUCUCCAUUUGGUCAUCTT-3′ (1023). They were all synthesized by Sangon Biotech (Shanghai, China). The PCR product of TDO2 was cloned into pEGFP-C1. The complete plasmid was verified by sequencing.

### 4.4. RNA-Immunoprecipitation (RIP)

#### 4.4.1. Obtaining the Cell Lysis Buffer

In line with the RIP kit instructions, the cells were collected into a centrifuge tube. After centrifugation at 1500 rpm for 5 min, the supernatant was discarded and the cells were collected. The cells were resuspended with the RIP lysate of the same volume as the cells, blown evenly, and then stood for 5 min. Each tube was filled with 200 µL of the cell lysate and stored at −80 °C.

#### 4.4.2. Preparation of Magnetic Beads

Two groups (IP and IgG) with magnetic beads were resuspended with 100 µL RIP wash buffer and incubated at room temperature for 20 min with about 10 µL anti-TDO2-Flag and IgG antibody. We placed the tubes on a magnetic rack and discarded the supernatant. We added 500 µL RIP wash buffer, discarded supernatant after vortex shock, and repeated the procedure one more time.

#### 4.4.3. RNA Binding Protein Immunoprecipitation

The tubes containing magnetic beads were put into the magnetic rack; the supernatant was discarded, and 900 µL RIP immunoprecipitation buffer was added into each tube. The cell lysates prepared in the first step were thawed rapidly and centrifuged at 14,000 rpm for 10 min to absorb 100 µL supernatant into 4.4.2 magnetic bead/antibody complex, resulting in a total volume of 1 mL. Meanwhile, 10 µL lysate was labeled as input and incubated for 3 h. After brief centrifugation, the tubes were placed on the magnetic rack and the supernatant was discarded. We added 500 µL RIP wash buffer, placed the EP tubes on the magnetic rack after vortex vibration, discarded the supernatant, and repeated the cleaning procedure three times.

#### 4.4.4. RNA Purification

The magnetic bead/antibody complexes were resuspended with 150 µL Proteinase K buffer and incubated at 55 °C for 30 min. We placed the tube on the magnetic stand and collected the supernatant into the new tubes. Then, 250 µL RIP wash buffer was added to each tube with the supernatant. We added 400 µL phenol, chloroform, and isoamyl alcohol to each tube, vortexed them for 15 s, and centrifuged at 14,000 rpm at room temperature for 10 min. We carefully absorbed the upper aqueous phase into a new EP tube; we added 50 µL salt solution I, 15 µL salt solution II, 5 µL precipitate enhancer, and 850 µL anhydrous ethyl alcohol (without RNase) to each tube and kept them overnight. After centrifuge at 14,000 rpm for 30 min, we carefully discarded the supernatant, rinsed with 80% ethanol once, and centrifuged at 14,000 rpm for 15 min. We discarded the supernatant carefully. The remaining RNA samples in the tubes were dried in the air. We dissolved them with 20 µL of DEPC water and stored them at low temperature.

#### 4.4.5. Library Construction and Detection

After the samples were confirmed to be qualified, the Small RNA Sample Pre Kit was used to construct the library. The special structure of the 3′ and 5′ terminals of the small RNA was used (the 5′ terminals have complete phosphate group, and the 3′ terminals have hydroxyl group), and the total RNA was employed as the starting sample to directly transfer the small RNA joint to both ends of RNA, and then reverse transcription was applied to synthesize cDNA (complementary DNA). After PCR (polymerase chain reaction) amplification, target DNA fragments were separated by PAGE gel electrophoresis. The cDNA library was obtained by gel cutting and recovery.

After the library was built, Agilent 2100 was used to test the library’s insert size. Then Hiseq/Miseq sequencing was performed for different libraries according to the effective concentration. The original image data obtained by Illumina Hiseq sequencing can be converted into sequence data through Base Calling to obtain the original sequencing data file. Starbase database. Available online: https://starbase.sysu.edu.cn/ (accessed on 3 December 2021). It was used to analyze the correlation between miR-126-5p and TDO2.

### 4.5. Luciferase Activity Reporter Assay

Target Scan 7.2. Available online: http://www.targetscan.org/vert_72/ (accessed on 3 December 2021) It was used to predict the potential target of miR-126-5p. The hsa-miR-126-5p-TDO2 WT/MUT in pSicheck-2 dual luciferase vector was constructed by inserting the target fragment TDO2-WT/MUT into pSicheck-2 as the skeleton vector. The template was derived from gene synthesis of TDO2-WT/MUT and confirmed by sequencing that it could be used as a template. XhoI and NotI were selected as insertion sites. 293T cells were cultured in 24-well plates, then co-transfected with miR-126-5p mimics or negative controls (Sangon Biotech, China) and WT or MUT reporter vector using Lipofectamine 3000. After 48 h, luciferase activity was measured using Dual-Glo Luciferase Assay System (Promega, cat: E1910). Each experiment was biologically repeated three times, and there were three technical replicates in each experimental run.

### 4.6. RNA Extraction and Quantitative Real-Time PCR (qRT-PCR)

Total RNA from the cultured cells was extracted using a Takara MiniBEST Universal RNA Extraction kit (Takara Scientific, Inc., Shiga, Japan) and quantified using Nanodrop-2000 (Thermo Fisher Scientific, Waltham, MA, USA). cDNA was synthesized from RNA using PrimeScript RT Reagent (Takara Scientific, Inc., Shiga, Japan). qRT-PCR was performed at ABI 7500 system ((Thermo Fisher Scientific, Waltham, MA, USA) using SYBR Premix Ex Taq II (Takara Scientific, Inc. Shiga, Japan). The primers used were as follows: PI3K forward, 5′-CTGCCTGCGACAGATGAGTGATG-3′ and reverse, 5′-ACTGCCCTATCCTCCGATT-ACCAAG-3′; AKT forward, 5′-TGACCATGAACGAGTTTGAGTA-3′, and reverse, 5′-GA-GGATCTTCATGGCGTAGTAG-3′; VEGF forward, 5′-ATCGAGTACATCTTCAAGCCA-T-3′ and reverse, 5′-GTGAGGTTTGATCCGCATAATC-3′; WNT1 forward, 5′-GAGAAA-CGGCGTTTATCTTCG-3′ and reverse 5′-GGATTCGATGGAACCTTCTGAG-3′; β-catenin forward, 5′-GGCTCTTGTGCGTACTGTCCTTC-3′ and reverse, 5′-CTTGGTGTCGGCTG-GTCAGATG-3′; TDO2 forward, 5′-GTCGACATGAGTGGGTGCCC-3′ and reverse 5′-GGATCCTTATTTGTCATCGTCATCCT-3′; β-actin forward, 5′-TCAAGATCATTGCT-CCTCCTG-3′, and reverse, 5′-CTGCTTGCTGATCCACATCTG-3′. The procedures were as follows: one cycle of 95 °C for 30 s, followed by 40 cycles of 95 °C for 5 s, and 60 °C for 34 s.

### 4.7. Cell Counting Kit-8 (CCK-8) Assay

CCK-8 assay was used to detect the cell proliferation rate. HCCLM3 cells were seeded in 96-well plates at a density of 2,000 cells/well. After incubation overnight, the cells were transfected with miR-126-5p inhibitor/mimic, inhibitor/mimic NC, pEGFP-C1-TDO2, and TDO2 siRNA. Subsequently, 10 μL CCK-8 reagent was added into each well and incubated for 1 h at 37 °C after transfection for the indicated times. The absorption value at 450 nm was detected on a microplate reader (Bio-Rad, Hercules, CA, USA).

### 4.8. Colony Formation Assays

After digestion and counting of logarithmic HCCLM3 cells, 200 cells/well were inoculated into six-well plates. After 12 h, the cells were transfected with miR-126-5p inhibitor/mimic, pEGFP-C1-TDO2, and TDO2 siRNA. The normal control group was assigned to two wells per group. The cells were placed in an incubator at 37 °C and 5% CO_2_ for 2 weeks, and the medium was changed every 4 days until visible cell colonies appeared. After fixing with formaldehyde, crystal violet was used for staining. Five fields were randomly selected for observation under an inverted microscope. The average cell mass number (greater than 50) was used as the colony count. The experiment was repeated three times.

### 4.9. Apoptosis Rate Detection (Annexin V-FITC/PI Double Staining)

HCCLM3 cells were transfected with miRNA-126-5p mimics, miRNA-126-5p inhibitor, TDO2, and siRNA for 48 h. The cells were digested by trypsin and collected after centrifugation. In accordance with the instructions of Annexin V-FITC/PI double-dyed cell apoptosis kit, precooled PBS at 4 °C was used. The cells were washed, and then 200 µL buffer, Annexin V-FITC, and PI 5 μL were added to each tube. After blending, the cells reacted at room temperature and away from light for 15 min. The experiment was repeated three times by flow cytometry.

### 4.10. Transwell Invasion Assay

Transwell invasion assays were used to detect the invasive and migratory abilities of the cells. First, 3 × 10^5^ cells were inoculated into a precoated supraventral Matrigel (BD Biosciences), and DMEM containing 10% FBS was added to the underlying Matrigel. After incubation for 24 h, noninvading cells were removed with cotton swabs, and the invading cells were fixed with methanol and stained with crystal violet. We then performed microscopy to count the number of the invading cells.

### 4.11. Wound Healing Assays

Exponentially grown cells were inoculated into six-well plates with 5 × 10^5^ cells/well; they were divided into five groups transfected with miR-126-5p inhibitor/mimic, pEGFP-C1-TDO2, and TDO2 siRNA. After 4 h, linear wounds were scratched with a 0.2-mL pipette tip. The dead cells were away washed with PBS. The medium was then replaced with DMEM containing 10% serum. Images were acquired at 0 and 48 h after the cells had been wounded. ImageJ (V1.8.0) software (National Institutes of Health, Bethesda, MD, USA) was used to measure the uncovered area of cells at each time point.

### 4.12. HPLC Detection of Tryptophan and Kynurenine Levels

The contents of tryptophan and kynurenine in cells were determined by HPLC. HCCLM3 cells were divided into the following five groups, with 1 × 10^6^ cells/well that were inoculated into six-well plates: control group, TDO2 group, miR-126-5p mimic group, miR-126-5p inhibitor group, and siRNA group. Then, 2 mL serum-free medium was added to each well. The culture medium was harvested after 48 h of incubation and then centrifuged and frozen until HPLC analysis. Standards of known concentrations of kynurenine and tryptophan were used for standardization of assays. Kynurenine and tryptophan reference standards were obtained from Solarbio Life Sciences (Beijing, China). Chromatographic separation conditions were described above.

### 4.13. Tumor Xenograft Assay

A mouse xenograft model was established in 5-week-old BALB/c-nu mice (Model Animal Research Institute of the Chinese Academy of Medical Sciences, Beijing, China) in accordance with the institutional guidelines. In brief, 5 × 10^6^ HCCLM3 cells suspended in 100 μL of PBS were injected subcutaneously into the left dorsal flanks of the mice. When the tumor size reached approximately 50 mm^3^, 9 mice were divided into the following three equal groups: group 126m as miR-126-5p agomir treatment, group 126i as miR-126-5p antagomir treatment, and group NC as blank control with PBS injection. Each tumor in the groups was injected with 50 μL solution containing 10 nmol miRNAs. After the treatments, the length (L) and width (W) of the tumors were measured every 3 days, and the mice were weighed every 4 days. Tumor volume (V) was calculated using the following equation: V = 0.5 × L × W^2^. On day 19, the mice were sacrificed and the tumors were dissected, weighed, and photographed. The tumor tissues were fixed in 4% paraformaldehyde for further studies.

### 4.14. Immunohistochemistry

The tumor tissues of the mice were fixed, embedded in paraffin, and sliced into consecutive tissue sections. Next, 5-μm-thick tissue sections were deparaffinized, dehydrated, and heated in citrate buffer (pH 6.0) for 15 min at 95 °C. To block the nonspecific bindings of the primary antibody, we added 1% bovine serum onto the slides for 20 min at room temperature. The paraffin sections of tumor tissues were taken. BAX (reflecting apoptosis), Ki-67 (reflecting proliferation), and VEGF (reflecting invasion) were detected by immunohistochemistry in line with the instructions of the SP kit. Finally, the sections were visualized with a DAB kit (ZSGB-bio, Beijing, China) and were counterstained with hematoxylin (Beyotime, Shanghai, China).

### 4.15. Statistical Analysis

SPSS software, version 18.0 (SPSS, Chicago, IL, USA) was used to analyze the data. The data were expressed as mean ± standard deviation (SD), and all the results were studied. *p* value < 0.05 was considered statistically significant. For comparison of two independent data sets, the Student’s t test was used.

## 5. Conclusions

In conclusion, our current study demonstrates that TDO2 is a direct target of miR-126-5p which regulated tumor progression via influencing the PI3K/AKT and WNT1 pathway. Furthermore, overexpression of miR-126-5p and TDO2 promoted HCCLM3 cells and HepG2 cells proliferation, migration, invasion and in vivo tumor cell xenograft formation and growth. These data suggest that miR-126-5p may be a novel therapeutic target for HCC and has independent prognostic value. In addition, miR-126-5p combined with TDO2 may be a promising tool for clinical prognostic assessment.

## Figures and Tables

**Table 1:** Comparison of the sRNA classification statistical table.

Types	TDO2_IP	TDO2_Input
Total	5855320	4249645
known_miRNA	146492	50017
rRNA	310336	126460
tRNA	2927643	1322479
snRNA	230579	420597
snoRNA	69119	46114
repeat	169	382
novel_miRNA	6473	11964

The comparison and annotation of all small RNAs with various types of RNAs were summarized. Total: The number of sRNA in reference sequence compared with each sample; known_miRNA: The sRNA number of known miRNA compared with each sample; rRNA/tRNA/snRNA/snoRNA: The sRNA number of rRNA, tRNA, snRNA and snoRNA; repeat: The sRNA number of repeat RNAs compared with each sample; novel_miRNA: The sRNA number of novel RNAs compared with each sample.

**Table 2:** Reads distribution across genomic regions.

Mapped_Region_Types	TDO2_IP	TDO2_Input
promoter	192790	159323
utr5	166823	242925
exon	219949	248064
intron	995928	1348570
utr3	97509	190775
Intergenic	3499178	2295842

The distribution statistics of sequenced sequences located on the reference genome were used to detect the origin of the sequenced sequences on the genome. The location region includes CDS, intergenic, intron region.

**Table 3:** The expression of the 19 known miRNAs matched.

miRNA	TDO2_IP	TDO2_Input
hsa-miR-126-5p	84	45
hsa-miR-140-5p	48	19
hsa-miR-200c-3p	19	2
hsa-miR-338-5p	6	1
hsa-miR-374c-5p	5	1
hsa-miR-155-5p	3	0
hsa-miR-4742-3p	2	1
hsa-miR-1277-5p	2	1
hsa-let-7c-3p	2	0
hsa-miR-217	2	0
hsa-miR-7974	1	0
hsa-miR-3655	1	0
hsa-miR-1266-5p	1	0
hsa-miR-3184-3p	1	0
hsa-miR-653-5p	1	0
hsa-miR-494-3p	1	0
hsa-miR-509-3-5p	1	0
hsa-miR-3929	1	0
hsa-miR-488-3p	0	1

Through Venn analysis and RIP experiment, we found 19 mirnas that already existed in the database. Their expression in the samples was shown in the table above.

## Data Availability

All the datasets on which the conclusions of the manuscript rely are presented in the paper.

## Supplementary Materials

The following are available online, known.miRNA.readscount, novel.miRNA.readscount, Target scan results.

## Sample Availability

Samples of the compounds are not available from the authors.
